# McConnell Sign in a Patient with Massive Acute Pulmonary Embolism

**DOI:** 10.1155/2011/201097

**Published:** 2011-07-05

**Authors:** Qaiser Shafiq, Ragheb Assaly, Yousuf Kanjwal

**Affiliations:** ^1^Department of Internal Medicine, The University of Toledo Medical Center, 3000 Arlington Avenue, Toledo, OH 43614, USA; ^2^Pulmonary and Critical Care Division, Department of Medicine, The University of Toledo Medical Center, 3000 Arlington Avenue, Toledo, OH 43614, USA; ^3^Cardiology Division, Department of Medicine, The University of Toledo Medical Center, 3000 Arlington Avenue, Toledo, OH 43614, USA

## Abstract

A 48-year-old female was admitted after experiencing a brief syncopal episode. Three weeks ago the patient sustained a right arm humerus bone fracture in a motor vehicle accident. Since the accident, her mobility has been limited. CT angiogram of the chest revealed massive bilateral pulmonary emboli. A 2D echocardiogram was performed, which demonstrated McConnell sign and severe right ventricle dysfunction. Considering potential of hemodynamic instability, the patient received fibrinolytic therapy with Alteplase. A subsequent 2D echocardiogram showed complete resolution of McConnell sign and right ventricle dysfunction.

## 1. Introduction

The severity of right ventricular dysfunction demonstrated by echocardiogram varies widely in patients with acute pulmonary embolism. We present a case of massive bilateral acute pulmonary embolism where an echocardiographic sign was demonstrated. 

## 2. Case History

**Figure 1:**
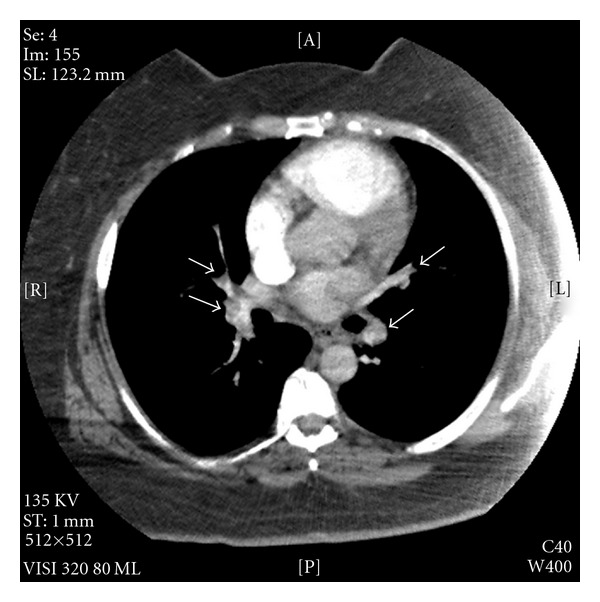
CT angiogram of the chest showing bilateral pulmonary emboli (arrows).

A 48-year-old African American woman was admitted an hour after experiencing a brief syncopal episode at her home. This episode was associated with profuse sweating and lightheadedness. According to the patient's daughter, no seizure-like activity was seen. Patient did not have past medical history of such episodes, seizures, stroke, venous thromboembolic disease, or myocardial infarction. Three weeks ago, she was involved in a motor vehicle accident and sustained a right arm humerus bone fracture and as a result her physical activities were limited since accident. On initial examination, she was hemodynamically stable but was experiencing lightheadedness and moderate respiratory distress due to the resting hypoxia. She required oxygen to maintain her oxygen saturation. No neurological deficits were found on further examination. Considering recent history of limited physical activity and resting hypoxia at the time of presentation, a CT angiogram of the chest was obtained to rule out acute pulmonary embolism which revealed bilateral large pulmonary emboli extending to all major branches (Figure 1).

**Figure 2:**
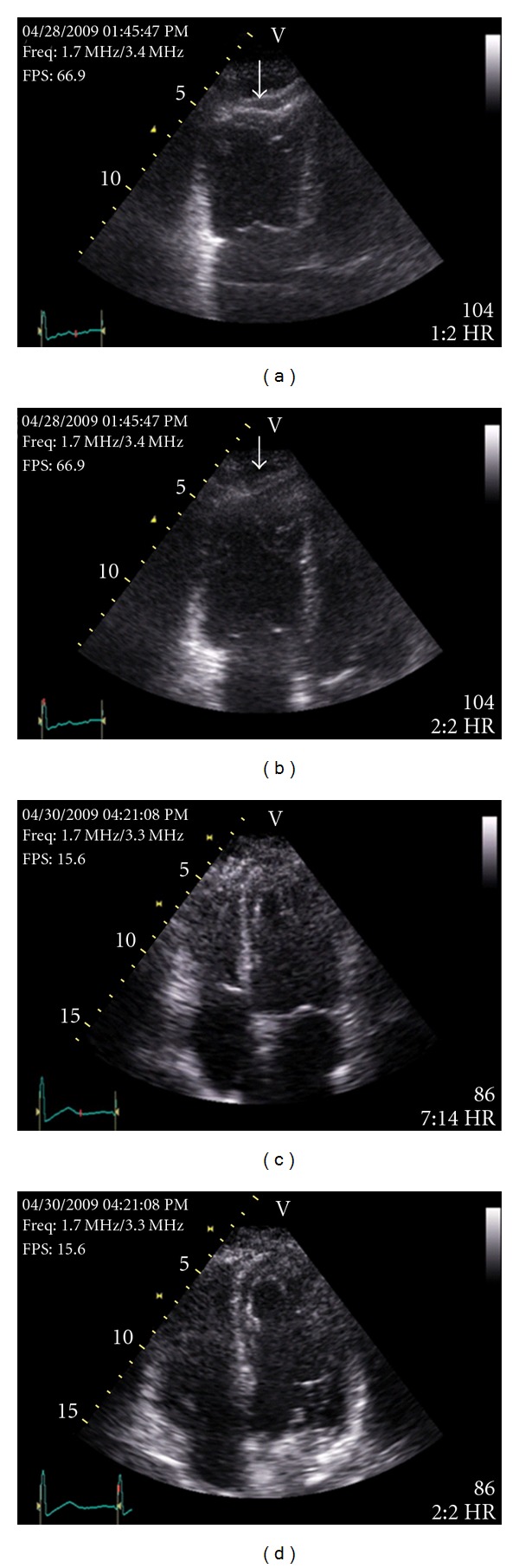
Prethrombolytic therapy transthoracic 2D echocardiogram showing marked hypokinesia of the right ventricle mid-free wall in end-systole (a) and end-diastole (b) while the right ventricle apex is spared (arrows). Postthrombolytic therapy transthoracic 2D echocardiogram showing complete resolution of right ventricular mid-free wall hypokinesia in end-systole (c) and end-diastole (d).

An immediate transthoracic 2D echocardiogram was performed to assess right ventricular function which demonstrated right ventricular dysfunction and a specific echocardiographic sign known as McConnell sign (Figures 2(a) and 2(b); Supplementary Video A available online at doi:10.1155/2011/201097). Considering such large bilateral pulmonary embolism and severe right ventricular dysfunction, fibrinolytic therapy with Alteplase was administered. In the next few hours, both the dyspnea and resting hypoxia improved. Two days later, a repeat 2D echocardiogram demonstrated complete resolution of right ventricular dysfunction and McConnell sign (Figures 2(c) and 2(d); Supplementary Video B).

## 3. Discussion

Right ventricular dysfunction in patients with acute pulmonary embolism indicates poor prognosis [1]. McConnell sign has 77% sensitivity and 94% specificity for the diagnosis of acute pulmonary embolism, with a positive predictive value of 71% and a negative predictive value of 96% [2]. However, recently, Casazza et al. demonstrated that McConnell sign can also be seen in cases of right ventricular infarction and thus cannot be considered as a specific marker for the diagnosis of acute pulmonary embolism [3]. In both cases, hypokinesia of the right ventricular mid-free wall is associated with right ventricular pressure overload [4]. Attempts to relate extent of pulmonary perfusion defects with regional right ventricular dysfunction has shown that at least 25% (moderate degree) of pulmonary perfusion defect is required for the McConnell sign to be demonstrated by transthoracic echocardiogram. Smaller perfusion defects have been noted to decrease acceleration time of the PA flow which could be a more sensitive parameter for assessment of right ventricle pressure overload in cases of small acute pulmonary embolism [5]. As noted above, our patient had massive bilateral pulmonary embolism which led to severe right ventricular mid-free wall dysfunction due to sudden increase in right ventricle pressure. Normalization of the regional right ventricle free wall dysfunction with thrombolysis has been shown in patients with massive pulmonary embolism as in our case [6]. This case report describes clinically useful echocardiographic sign for the diagnosis of massive pulmonary embolism. Recognition of this sign could help critical care physicians in early decision making including thrombolysis.

## 4. Clinical Pearls

1. 2D echocardiography is a useful supportive tool to diagnose acute pulmonary embolism.
2. McConnell sign indicates at least a moderate-size pulmonary perfusion defect which may portend significant hemodynamic instability leading to death. Close monitoring is mandatory in such cases.

## Supplementary Material

Video A: McCconnell sign prethrombolytic.
Video B: Resolution of McConnell sign postthrombolytic.
